# Supplementary material for: Focal neural perturbations reshape low-dimensional trajectories of brain activity supporting cognitive performance
Source: Nat Commun. 2022 Jan 10;13:4. doi: 10.1038/s41467-021-26978-2 (PMC8749005; doi:10.1038/s41467-021-26978-2)
Supplement: Supplementary file 1 — Supplementary Information [file 41467_2021_26978_MOESM1_ESM.pdf]

Focal neural perturbations reshape low-dimensional trajectories of brain activity supporting cognitive performance

Kartik K. Iyer[1*], Kai Hwang[2, 3], Luke J. Hearne[1], Eli Muller[4], Mark D'Esposito[3], James M. Shine[4], Luca Cocchi[1*]

[1]QIMR Berghofer Medical Research Institute, Brisbane, QLD 4006, Australia
[2]Department of Psychological and Brain Sciences and The Iowa Neuroscience Institute, The University of Iowa, Iowa City, IA 52242, USA
[3]Helen Wills Neuroscience Institute, University of California, Berkeley, CA 94720-1650, USA.
[4]The University of Sydney, Sydney, NSW 2050, Australia

Supplementary Table and Figures

	Baseline 1-back (%)	*Baseline 2-back*	*S1 1-back*	*S1 2-back*	*iPS 1-back*	*iPS 2-back*
PHATE1	82.75	82.18	86.11	81.90	67.47	76.72
PHATE2	13.08	13.68	6.73	15.17	31.22	20.64
PHATE3	3.18	2.93	5.59	1.64	1.13	2.02
PHATE4	0.44	0.80	1.06	0.92	0.08	0.35
PHATE5	0.29	0.20	0.28	0.17	0.05	0.14

Supplementary Table 1. *Explained variance of PHATE components as captured by a denoised affinity-based transition embedding space.* This table summarizes the variance explained (%) by the top five PHATE dimensions. The first three dimensions explain >98% of the variance in the data. The variance explained by the three first dimensions remain consistent for the baseline and the S1 sessions, across 1-back and 2-back conditions. In contrast, TMS on iPS causes a shift in the overall variance captured by the PHATE dimensions (particularly the first and second dimensions). See Methods and Moon et al.[12] for further details.

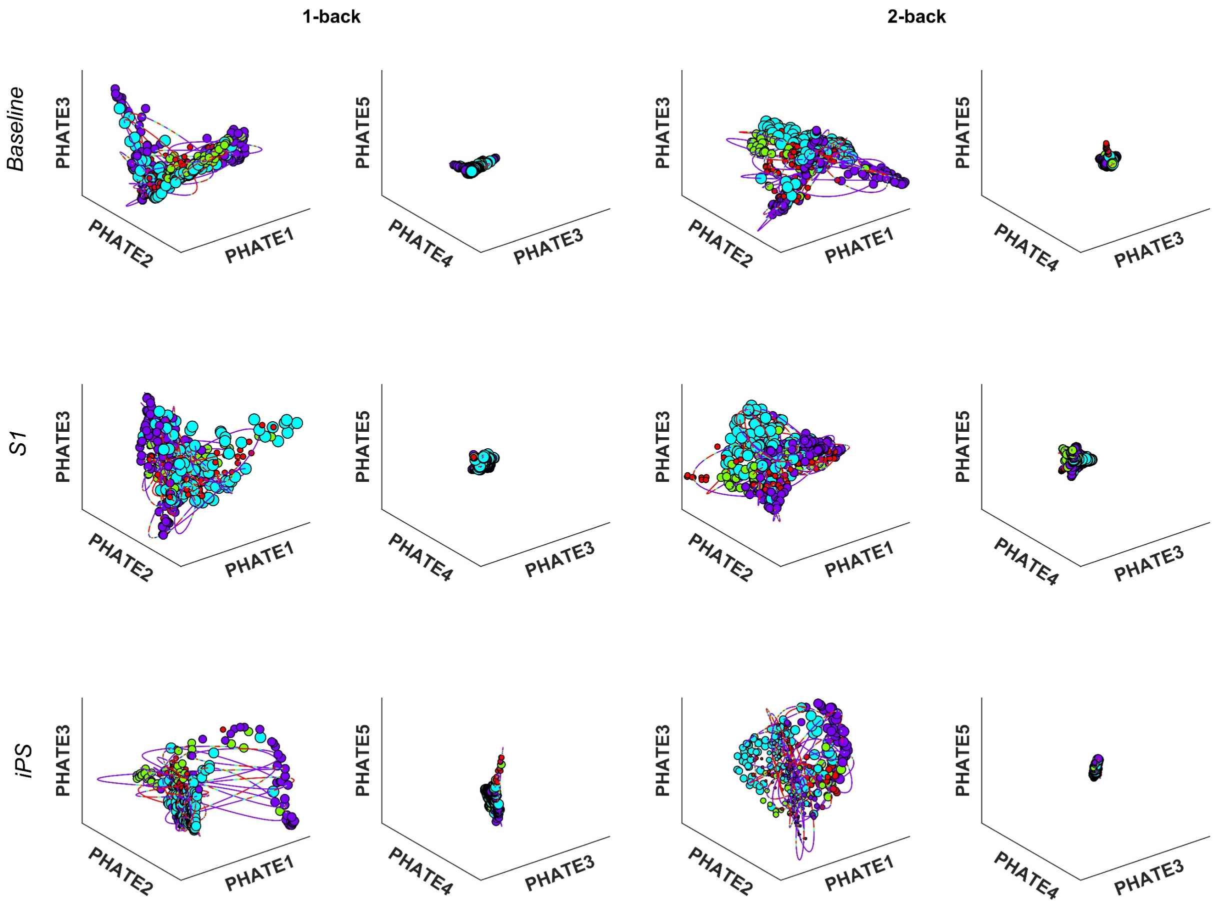

Supplementary Figure 1. *Session and working memory load effects on PHATE4 and PHATE5.* The figure highlights the contribution of low-dimensional trajectories defined by the first three dimensions (PHATE1, PHATE2, PHATE3) as a function of working memory load (‘correct’ responses from trial conditions shown). Low-dimensional trajectories defined by control dimensions (PHATE4 and PHATE5) remain unchanged for ‘correct’ responses in the same trial conditions. Source data are provided as a Source Data file.

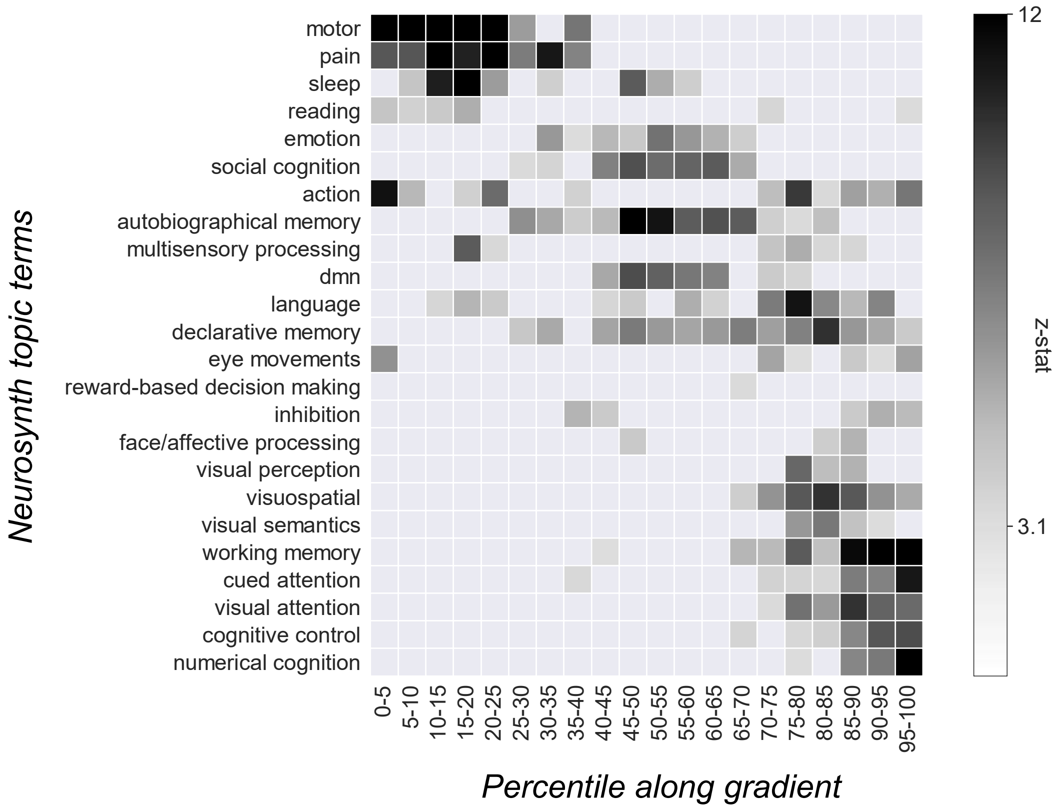

Supplementary Figure 2. *High-dimensional projections of the low-dimensional embedding linked to associative-cognitive processes defined by meta-analytic data (NeuroSynth).* This figure highlights the association of the overall spatial weights (average across PHATE1, PHATE2, PHATE3 dimensions for correct 2-back trials, all experimental sessions) with 24 NeuroSynth topic terms aggregated from over 5000 neuroimaging studies. The figure shows that spatial weights estimated from low-dimensional brain activity were highly associated with cognitive processes such as working memory and numerical cognition.

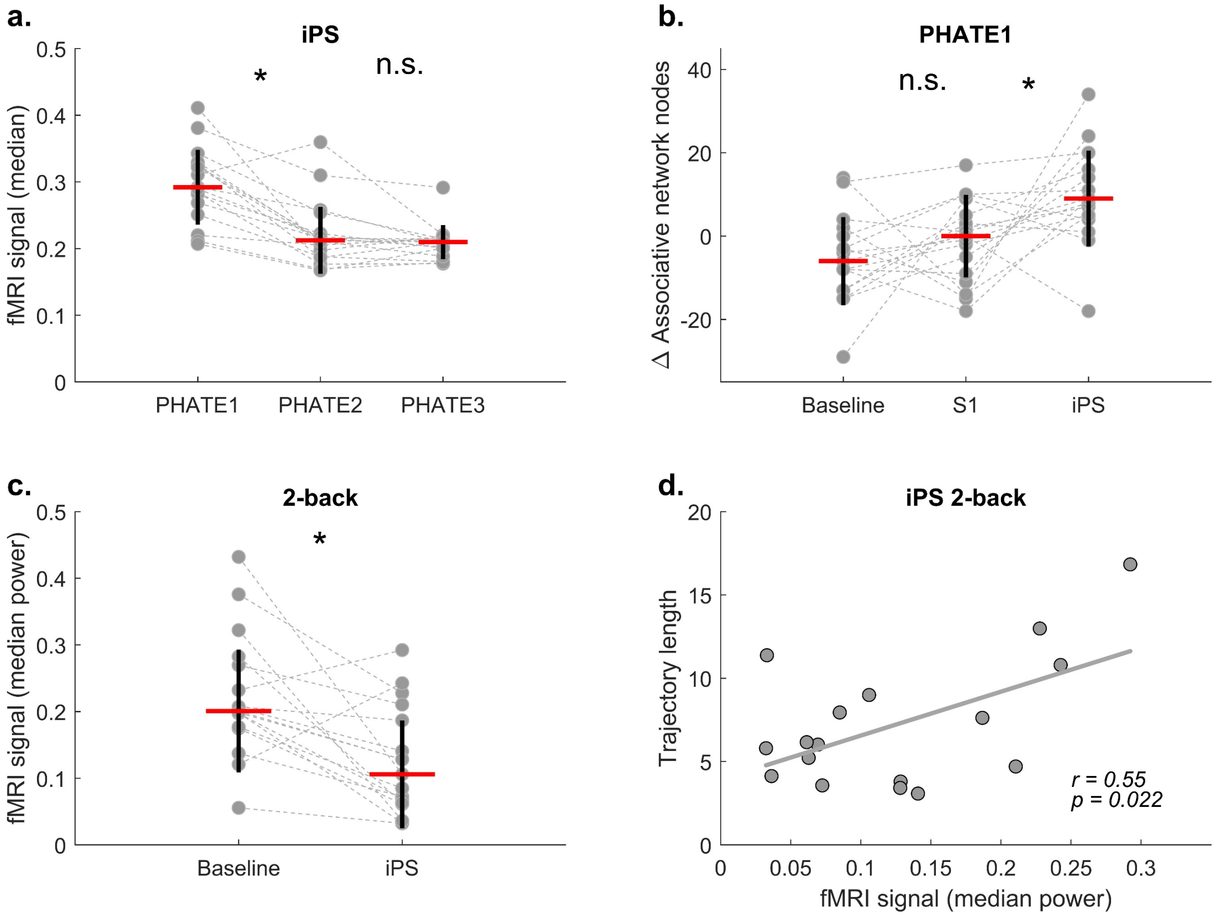

Supplementary Figure 3. *System-level and local-level changes for correct 2-back trials following stimulation of iPS*. **a,** For median fMRI signals recorded during the iPS session, a within-subject ANOVA showed a significant effect ($F_{(2,\ 48)}$=18.5, *p*=$1.1x10^{-6}$; *n*=17 examined within the iPS session across PHATE1, PHATE2 and PHATE3 dimensions; median represented by red line; within-dimension S.D. shown with black lines). No significant differences in fMRI signal across PHATE dimensions were detected for baseline and S1 sessions (*p>0.3*; not shown). The effect detected in the iPS session was driven by increased fMRI signal in key brain regions (top 20%) contributing to PHATE1 compared to fMRI signal in key regions contributing to PHATE2 and PHATE3 (paired t-test, PHATE1 > PHATE2 p_{FDR}=$8.4x10^{-5}$, PHATE1 > PHATE3 p_{FDR}=$2.1x10^{-6}$, and PHATE2 versus PHATE3 p_{FDR}=*0.18;* two-sided test). **b,** Analysis of changes in brain regions attributed to the associative network (cyan in Fig.1b) as a function of working memory load and session showed a significant interaction ($F_{(2,\ 96)}$=8.64, *p*=$3.5x10^{-4}$, *n*=17 examined over the three experimental sessions). Positive values in the panel indicate recruitment of brain regions (number of nodes) comprising the associative network in the 2-back condition compared to 1-back condition. That is, the interaction was driven by an increased number of brain regions contributing to PHATE1 in correct 2-back trials (paired t-test, iPS > baseline p_{FDR}=*0.0007*, iPS > S1 p_{FDR}=*0.027*, and baseline versus S1 p_{FDR}=*0.26;* two-sided test). No significant differences were detected in all other network communities. Within-session median values represented by red lines and within-session S.D. shown with black lines. **c,** The fMRI signal (median power) of iPS was reduced in the iPS session (correct 2-back trials) compared to the baseline session (paired t-test, *p=0.0023* two-sided test*, n*=17 examined between the baseline and iPS sessions). Each dot in the panel represents a participant (within-session S.D. shown with black lines). **d**, Pearson's correlation between iPS fMRI signal (2-back condition, correct trials) and length of the low-dimensional trajectory. Individuals with lower task-induced fMRI signal in iPS (following cTBS) showed shorter low-dimensional trajectories (*r=0.55*, *p=0.022,* two-tailed) and higher 2-back accuracy (see Fig. 3b). For all panels, asterisks (*) indicate statistical significance, adjusting for multiple comparisons where appropriate. Source data are provided as a Source Data file.

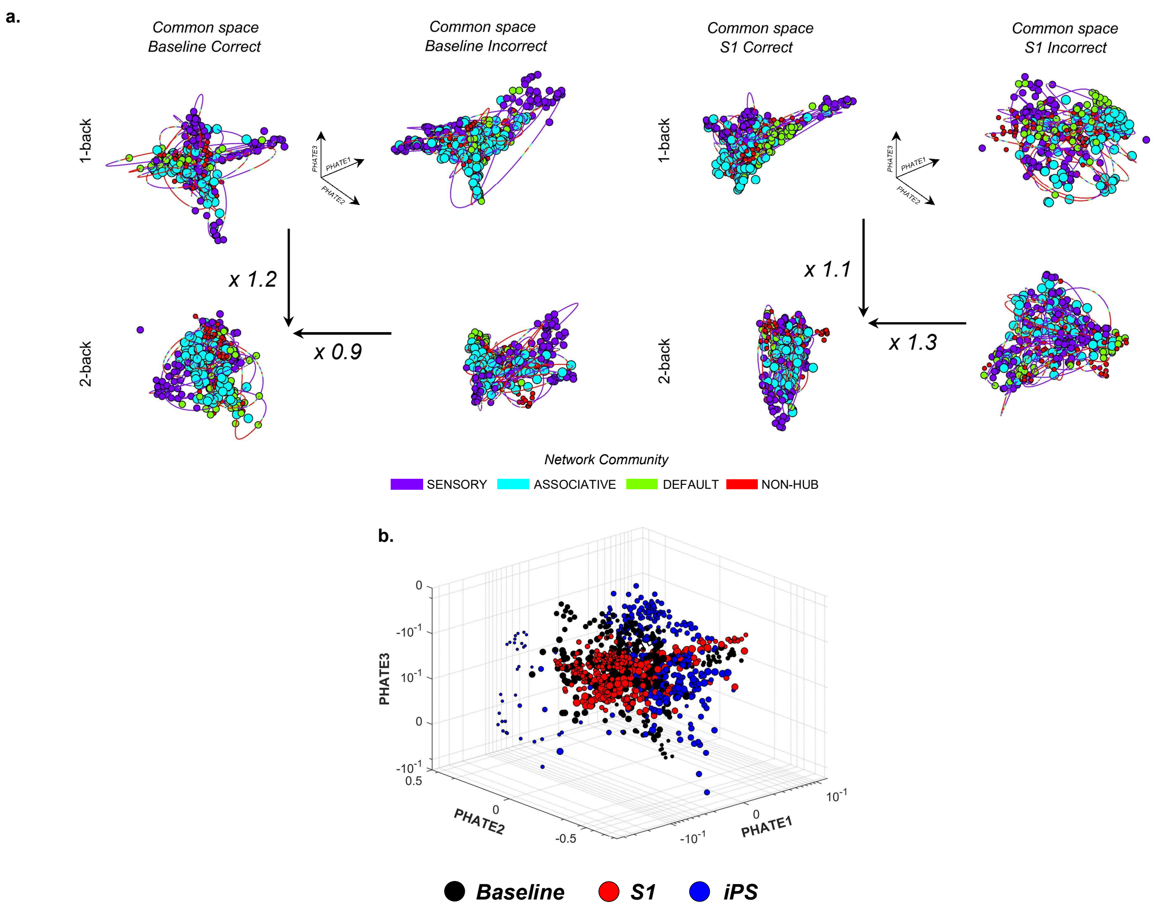

Supplementary Figure 4. *Low-dimensional trajectories for baseline (no TMS) and S1 (following TMS on S1) sessions in the common embedding space*. **a,** Results suggest that a measured degree of expansion (that does not reach statistical significance) of low-dimensional trajectories in the task embedding space is required to successfully accommodate an increase in working memory load in both baseline and S1 sessions. **b,** Representation of low-dimensional trajectories for 1-back correct trials in the common embedding space for baseline, S1 and iPS sessions (visualized in log space, same embedding space across experimental sessions). Source data are provided as a Source Data file.
