# Supplementary material for: Focal neural perturbations reshape low-dimensional trajectories of brain activity supporting cognitive performance
Source: Nat Commun. 2022 Jan 10;13:4. doi: 10.1038/s41467-021-26978-2 (PMC8749005; doi:10.1038/s41467-021-26978-2)
Supplement: Supplementary file 3 — Reporting summary [file 41467_2021_26978_MOESM3_ESM.pdf]

nature portfolio

Corresponding author(s): Kartik Iyer
Luca Cocchi

Last updated by author(s): Oct 15, 2021

# Reporting Summary

Nature Portfolio wishes to improve the reproducibility of the work that we publish. This form provides structure for consistency and transparency in reporting. For further information on Nature Portfolio policies, see our Editorial Policies and the Editorial Policy Checklist.

## Statistics

For all statistical analyses, confirm that the following items are present in the figure legend, table legend, main text, or Methods section.

| n/a | Confirmed | |
|---|---|---|
| ☐ | ☒ | The exact sample size ($n$) for each experimental group/condition, given as a discrete number and unit of measurement |
| ☐ | ☒ | A statement on whether measurements were taken from distinct samples or whether the same sample was measured repeatedly |
| ☐ | ☒ | The statistical test(s) used AND whether they are one- or two-sided<br>*Only common tests should be described solely by name; describe more complex techniques in the Methods section.* |
| ☐ | ☒ | A description of all covariates tested |
| ☐ | ☒ | A description of any assumptions or corrections, such as tests of normality and adjustment for multiple comparisons |
| ☐ | ☒ | A full description of the statistical parameters including central tendency (e.g. means) or other basic estimates (e.g. regression coefficient) AND variation (e.g. standard deviation) or associated estimates of uncertainty (e.g. confidence intervals) |
| ☐ | ☒ | For null hypothesis testing, the test statistic (e.g. $F$, $t$, $r$) with confidence intervals, effect sizes, degrees of freedom and $P$ value noted<br>*Give P values as exact values whenever suitable.* |
| ☒ | ☐ | For Bayesian analysis, information on the choice of priors and Markov chain Monte Carlo settings |
| ☒ | ☐ | For hierarchical and complex designs, identification of the appropriate level for tests and full reporting of outcomes |
| ☐ | ☒ | Estimates of effect sizes (e.g. Cohen's $d$, Pearson's $r$), indicating how they were calculated |

*Our web collection on statistics for biologists contains articles on many of the points above.*

## Software and code

Policy information about availability of computer code

| | |
|---|---|
| Data collection | For fMRI data preprocessing, FMRIPREP v1 and ANTS v2.1.0 were used. fMRI outputs generated from these processing pipelines were used for data analysis. |
| Data analysis | Custom code written in MATLAB2020b were used for the analysis of fMRI outputs generated. A github link houses all necessary data required to run and reproduce data analysis presented within the manuscript: https://github.com/kneuro/trajectories_perturbations |

For manuscripts utilizing custom algorithms or software that are central to the research but not yet described in published literature, software must be made available to editors and reviewers. We strongly encourage code deposition in a community repository (e.g. GitHub). See the Nature Portfolio guidelines for submitting code & software for further information.

## Data

Policy information about availability of data

All manuscripts must include a data availability statement. This statement should provide the following information, where applicable:

- Accession codes, unique identifiers, or web links for publicly available datasets
- A description of any restrictions on data availability
- For clinical datasets or third party data, please ensure that the statement adheres to our policy

Data availability. All data generated and evaluated are available on the following GitHub repository: https://github.com/kneuro/trajectories_perturbations. The GitHub repository has the following DOI: https://doi.org/10.5281/zenodo.5568470. The repository contains all data required to reproduce the results. The raw and preprocessed fMRI data are available from the authors upon reasonable request. The web interface to the NeuroSynth database is available here: www.neurosynth.org. A reporting summary for this Article is available as a Supplementary Information file. Source data are provided with this paper

# Field-specific reporting

Please select the one below that is the best fit for your research. If you are not sure, read the appropriate sections before making your selection.

☐ Life sciences ☒ Behavioural & social sciences ☐ Ecological, evolutionary & environmental sciences

For a reference copy of the document with all sections, see nature.com/documents/nr-reporting-summary-flat.pdf

# Behavioural & social sciences study design

All studies must disclose on these points even when the disclosure is negative.

**Study description**

The study uses quantitative methods. These quantitative methods are applied on fMRI data and involve data reduction and manifold embedding techniques known as the potential of heat diffusion for affinity-based transition embedding algorithm (PHATE, see Moon et al (2019) cited 12th in Reference list).

**Research sample**

A total sample of 17 human participants was included in the final analysis. This sample was a healthy adult group aged between 18 and 35 years of age, with a split of 11 females and 6 males. The sample was chosen by convenience and is representative with regard to the purpose of this research.

**Sampling strategy**

This study employed a randomized, within-subjects design, wherein 17 human participants completed 3 experimental sessions. No sample size calculations were explicitly performed.

Sample sizes were deemed sufficient due to previously published fMRI, TMS studies by co-authors (D'Esposito, M):

Gratton, C., Lee, T. G., Nomura, E. M., & D'Esposito, M. (2013). The effect of theta-burst TMS on cognitive control networks measured with resting state fMRI. Frontiers in systems neuroscience, 7, 124.

Lee, T. G., & D'Esposito, M. (2012). The dynamic nature of top-down signals originating from prefrontal cortex: a combined fMRI–TMS study. Journal of Neuroscience, 32(44), 15458-15466.

Lee, T. G., Blumenfeld, R. S., & d'Esposito, M. (2013). Disruption of dorsolateral but not ventrolateral prefrontal cortex improves unconscious perceptual memories. Journal of neuroscience, 33(32), 13233-13237.

Effect sizes were not known prior to analysis, however based on a within-subjects design of 17 participants with completed TMS and fMRI a small-to-moderate effect size was estimated.

**Data collection**

Each experimental session involved the completion of a baseline session first, followed by a short course of non-invasive brain stimulation (TMS) with immediate participation of a cognitive task (within 10 minutes of receiving stimulation) in an MRI scanner on separate days. The TMS device used was a MagStim Super Rapid 2 stimulator with a 70 mm diameter figure-eight double air film coil. The TMS protocol applied was a continuous theta-burst stimulation (cTBS) protocol. MRI data in participants was acquired using a Siemens 3T Tim/Trio scanner at the Henry H. Wheeler, Jr. Brain Imaging Center at the University of California, Berkeley. All participants completed a working memory (n-back) task.

For these sessions, the main experimenter (Hwang, K) was not blinded to the study hypothesis. A research assistant was also present in all experimental sessions alongside the main experimenter.

**Timing**

Data collection was undertaken in February 2017 and was completed in December 2017

**Data exclusions**

No data was excluded from the final sample of 17 participants analyzed.

**Non-participation**

A total of 5 subjects were excluded due to non-participation issues. 2 subjects were excluded because of hardware malfunction. Additionally, 2 subjects were excluded because of they fell asleep in the scanner. 1 other subject dropped out of the experiment and declined to continue. Following participant exclusions, 17 participants out of a possible 22 participants had been identified with full data collected across all 3 experimental sessions.

**Randomization**

Following a baseline MRI session, the order of the TMS sessions across participants were randomized. The presentation of trials within the working memory n-back task were also presented in a randomized fashion across all experimental sessions.

# Reporting for specific materials, systems and methods

We require information from authors about some types of materials, experimental systems and methods used in many studies. Here, indicate whether each material, system or method listed is relevant to your study. If you are not sure if a list item applies to your research, read the appropriate section before selecting a response.

## Materials & experimental systems

| n/a | Involved in the study |
| --- | --- |
| ☒ | ☐ Antibodies |
| ☒ | ☐ Eukaryotic cell lines |
| ☒ | ☐ Palaeontology and archaeology |
| ☒ | ☐ Animals and other organisms |
| ☐ | ☒ Human research participants |
| ☒ | ☐ Clinical data |
| ☒ | ☐ Dual use research of concern |

## Methods

| n/a | Involved in the study |
| --- | --- |
| ☒ | ☐ ChIP-seq |
| ☒ | ☐ Flow cytometry |
| ☐ | ☒ MRI-based neuroimaging |

# Human research participants

Policy information about studies involving human research participants

| | |
| --- | --- |
| Population characteristics | We collected data from a sample of healthy adult participants (aged 18–35, 11 females, 6 males) |
| Recruitment | Subjects were recruited by flyers and emails sent to the Berkeley community, and participants were signed up by the order in which they contacted the recruitment contact provided. No sampling bias (self-selection or otherwise) to report for this study. |
| Ethics oversight | Committee for the Protection of Human Subjects at the University of California, Berkeley. Written consent was obtained for all participants. |

Note that full information on the approval of the study protocol must also be provided in the manuscript.

# Magnetic resonance imaging

## Experimental design

| | |
| --- | --- |
| Design type | Task block/event design |
| Design specifications | Across the three neuroimaging sessions, participants were presented a sequential set of pictures randomly selected from a set of 120 pictures of human faces and buildings. The key experimental manipulation involved the modulation of working memory load (2-back versus 1-back). Each fMRI run comprised trials for each task condition (1- or 2-back). At the start of each run, participants viewed a fixation cross for 3 seconds, followed by counterbalanced n-back task blocks interleaved with a rest task block (fixation cross). Each n-back task block started with a 2-second initiation cue, followed by 13 trials of stimuli. Each trial started with an image displayed at center of the screen for 0.5 seconds, followed by a randomly jittered intertrial fixation that lasted 1.5 to 10 seconds. Two to four repetitions of both stimuli conditions were used within each task block, with presentation sequences randomized separately. For the first five participants, the task comprised runs of 155 seconds (two 60-second n-back blocks interleaved with a 25-second rest block and 7 seconds of a final fixation). The remaining 12 participants undertook slightly longer runs (236 TRs per run), with each run consisting of three 60-second task blocks interleaved with two 30-second rest blocks and a 10-second final fixation. The total number of trials across the three sessions for all participants was identical (78 trials for each n-back condition). |
| Behavioral performance measures | Participants were required to make a button press to identify the category of the picture presented, wherein a correct or incorrect button press was logged across all 78 trials. The total number of correct and incorrect trials were then used to formulate overall task accuracy for 1-back and 2-back trials respectively. Expected performance was ensured across experimental sessions - please see Hwang et al. 2020 (Cerebral Cortex) for details. |

## Acquisition

| | |
| --- | --- |
| Imaging type(s) | fMRI, T1 |
| Field strength | 3T |
| Sequence & imaging parameters | A 32-channel head coil with a multiband echo-planar imaging sequence (acceleration factor=4, TR=1s; TE=33.2 ms; flip angle=40º; voxel size: 2.5 mm3 isotropic voxels with 52 axial slices). Structural MRI data were also acquired: TR=2530 ms; TE=1.64/3.5/5.36/7.22 ms; flip angle=7º; field of view=256 x 256, 176 sagittal slices, 1 mm3 voxels; 2x GRAPPA acceleration. |
| Area of acquisition | Whole-brain scan |
| Diffusion MRI | ☐ Used ☒ Not used |

## Preprocessing

| | |
| --- | --- |
| Preprocessing software | FMRIPREP v1 and ANTS v2.1.0. Custom matlab scripts, which are available at https://github.com/kneuro/trajectories_perturbations |

| | |
|---|---|
| Normalization | Data was normalized to the ICBM 152 Nonlinear Asymmetric template version 2009c via nonlinear registration (ANTS v2.1.0). Within FMRIPREP, FSL's MCFLIRT routine and registered to T1 images using boundary-based registration. |
| Normalization template | ICBM152 Nonlinear Asymmetric template version 2009c |
| Noise and artifact removal | fMRI data was spatially smoothed using a 4 mm full-width-at-half-maximum Gaussian Kernel, and a nuisance regression was performed (AFNI's 3dDeconvolve) to remove linear drifts, signals from six rigid-body motion parameters and their temporal derivatives. The averaged signal from white matter and ventricles were also removed. |
| Volume censoring | Within FMRIPREP, motion confounds were minimized by removing all fMRI volumes (prior to regression analyses) that exceeded framewise displacement greater than 0.2 mm. |

## Statistical modeling & inference

| | |
|---|---|
| Model type and settings | A quantitative dimensionality-reduction method (Potential of Heat diffusion for Affinity based Transition Embedding, described in Moon et al. 2019 (Nature Biotechnology) was used to analytically model data. This method utilizes information geometry and manifold learning to derive local and global structures that exist within high-dimensional data |
| Effect(s) tested | Within-subjects ANOVA and t-tests were used to compare manifold trajectories as a function of working memory load (1-back versus 2-back) and performance (correct versus incorrect trials). For bivariate correlations, the Pearson correlation coefficient was used. The D1 method byWilcoxon & Tian 2008 (The Journal of General Psychology) was used to compare dependent correlations (median length of manifold trajectories with overall 2-back accuracy) across sessions. |

Specify type of analysis: ☐ Whole brain ☒ ROI-based ☐ Both

Anatomical location(s): The Gordon parcellation (333 regions) were used for region based approximations, see Gordon et al 2016 (Cerebral Cortex)

| | |
|---|---|
| Statistic type for inference (See Eklund et al. 2016) | No voxel-wise or cluster-based statistics were required for this analysis. |
| Correction | FDR corrections, via the Benjamini-Hochberg procedure were applied to statistical procedures. |

## Models & analysis

| n/a | Involved in the study |
|---|---|
| ☒ | ☐ Functional and/or effective connectivity |
| ☒ | ☐ Graph analysis |
| ☒ | ☐ Multivariate modeling or predictive analysis |
